# The impact of first wave of COVID-19 on the nursing-sensitive and rehabilitation outcomes of patients undergoing hip fracture surgery: a single centre retrospective cohort study

**DOI:** 10.1186/s12912-022-00848-8

**Published:** 2022-03-25

**Authors:** M Morri, E Ambrosi, D Raffa, R Raimondi, A Evangelista, A Mingazzini, C Forni

**Affiliations:** 1 IRCCS Istituto Ortopedico Rizzoli, Servizio Di Assistenza Infermieristico Tecnico E Riabilitativo Bologna, Bologna, Italia; 2 Dipartimento Di Diagnostica E Sanità Pubblica, Università Degli Studi Di Verona, Verona, Italia; 3 Laboratorio Di Emodinamica, Ospedale Maggiore, Bologna, Italia

**Keywords:** COVID-19, Nursing-sensitive outcome, Rehabilitation, Hip fracture

## Abstract

**Background:**

During the COVID-19 pandemic, the care of hip fracture patients remains a clinical priority. To date, there is limited empirical knowledge about the impact of pandemic on the care of patients surgically treated for hip fracture, affected or not by COVID-19.

**Objective:**

To investigate the effects of the COVID-19 pandemic on the nursing-sensitive and rehabilitation outcomes of frail patients undergoing hip fracture surgery.

**Methods:**

A retrospective cohort study was conducted in an Italian Orthopaedic Research Institute. All patients aged ≥ 65 years admitted with fragility hip fractures between 1st March and 30th June in 2019 (group PP: pre-pandemic) and in the same period in 2020 (group P: pandemic), were compared. In the P group, COVID-19 positive patients were excluded due to the presence of a specific treatment pathway. Data on patient demographics and baseline characteristics, and peri-operative care factors were obtained from the Institute’s computer-based patient-record system. The primary outcome was the incidence of any stage hospital-acquired pressure ulcers (PUs). The secondary outcome was time to first static verticalization and to first ambulation.

**Results:**

Three-hundred and sixty patients were included in the study, which comprised 108 patients in PP group and 252 patients in P group. Overall PUs incidence was significantly higher in the P-group (21.8%) than in the PP-group (10.2%) (*p* = 0.009). Specifically, the incidence of sacral PUs was significantly lower in P-group (38.1%) vs PP-group (91%) (*p* = 0.004); on the contrary, the incidence of PUs localized to the heels or other body sites were significantly higher in P-group (30.9% and 30.9%, respectively) vs PP-group (0% and 9%, respectively) (*p* = 0.004).

No significant between groups differences were found for all the secondary outcomes.

**Conclusion:**

In the pandemic period, nursing and rehabilitation care provided to patients with fragility hip fracture maintained high standards comparable to the pre-pandemic period. The increase in PUs incidence in the pandemic period was probably due to the older age of the patients admitted to hospital. The qualitative evaluation of the care administered and the emotional impact of the pandemic on the patients are very interesting topic which would deserve further investigation.

## Background

The global health emergency due to the spread of COVID-19 has caused unprecedented pressure on the health systems at all levels [1]. COVID-19 has had a direct impact on the health status of populations due to the extremely high numbers of Intensive Care Unit (ICU) admissions and deaths [2, 3], but it has also resulted in several equally strong indirect consequences in different fields, even in patients not affected by COVID [4–7]. In order to cope with the great demand of health resources, hospitals have had to invest human and physical resources, redirecting them from other care activities [8]. Aiken et al. [9] highlighted how the redirection of nurses to COVID units, which resulted in the reduction of nurses involved in standard care, as well as the employment of nursing personnel lacking context-specific clinical skills due to the reorganization of care, are to be correlated with an increase in complications such as pressure ulcers, falls and urinary tract infections, and mortality, in any type of patient. Stifer et al. [10] highlight the importance of focusing attention on the "Nurse-Sensitive Indicators" in order to evaluate the patient care quality.

In the first phase of spread of the COVID pandemic, all non-essential orthopaedic surgical interventions were postponed, at the same time the decision to create ad hoc pathways to manage essential interventions ensured the provision of emergency care during the pandemic. This organizational model has led to a reduction in the overall number of hospitalized patients, although the number of patients surgically treated for fragility hip fracture has not decreased [11–13].

Fragility fractures are associated with higher chances of clinical worsening in terms of quality of life and disability in the medium and long term [14]. Following this traumatic event, more than half of the patients are unable to recover pre-fracture motor skills such as walking ability [15]. Considering the pandemic impact on patient care, data on patients surgically treated for hip fracture, either COVID-19 positive or not, are lacking.

The aim of this study was therefore to investigate the effects of the COVID-19 pandemic on nursing-sensitive and rehabilitation outcomes of frail patients undergoing hip fracture surgery.

## Methods

### Study design, setting and sample

A retrospective cohort study was conducted in an Orthopaedic Research Institute in the centre-north of Italy. All patients aged 65 years or older admitted with fragility hip fractures between 1^st^ March and 30^th^ June in 2019 (group PP: pre-pandemic) and in 2020 (group P: pandemic) were compared. Those patients with diaphyseal or pathological fractures, and/or did not sign a consent at hospital admission giving permission for their data to be used in the future, were excluded. Additionally, for the 2020 cohort patients with a diagnosis of COVID were also excluded, due to the different treatment characteristics.

The study was approved by the Institute’s Ethics Committee CE AVEC: 27/2021/Oss/IOR. The research protocol was registered on ClinicalTrials.gov (NCT04882670).

### Patient and public involvement statement

Patients or the public were not involved in the design, or conduct, or reporting, or dissemination plans of our research.

### Standard care

#### Hip fracture surgery

Early surgery within 48 h from the trauma was generally guaranteed. It could be postponed just for medical reasons, such as the need to stop anticoagulants or to stabilize clinical conditions. Patients were admitted either to an orthopaedic surgery department or to an orthogeriatric department, depending on beds’ availability. The duty orthopaedic surgeon, based on the patient’s age, clinical conditions and type of fracture, established the surgical technique.

#### Pressure ulcers (PUs) preventive care

All patients were assessed for PU risk through the Braden Scale [16] at hospital admission, and, if the Braden score was < 17, they were placed on a higher‐specification foam or a dynamic anti-decubitus mattress within 24 h. Staff nurses assessed patient pressure points at least daily and guaranteed routine positioning every four hours after surgery in order to minimize friction and shear. Skin care was carried on according to EPUAP guidelines [17].

#### In-hospital rehabilitation

The inpatient rehabilitation treatment started one day after surgery. It consisted of two 30-min physiotherapy sessions a day from Monday to Saturday aiming at obtaining an early sitting and standing position and then walking.

#### Post-hospital rehabilitation

After the hospitalisation phase, a pathway was defined for each patient tailored to their rehabilitation and care needs and community resource availability.

Timing and techniques of hip fracture surgery, PUs preventive care and in-hospital rehabilitation treatments have been the same in the PP and P periods.

### Hospital reorganization during pandemic

During the COVID-19 pandemic, our hospital, which is usually devoted to the management of complex orthopaedic surgical cases, including tumor, infections, and revision surgery, with trauma cases normally representing less than 30% of its overall surgical activity, became the reference center for the treatment of orthopaedic emergencies in one north-eastern Italian city with about 400.000 inhabitants. Thus, an extensive rearrangement of surgical activity and clinical protocols was undertaken in order to support this change. Moreover, all the procedures aimed at preventing and containing the spread of COVID-19 were put in place according to available national and international guidelines [18, 19]. The activity related to elective surgeries was suspended throughout the hospital; thus, four out of eleven surgical units, such as those dedicated to reconstructive orthopaedic surgery, shoulder and elbow surgery, private surgery, and post-operative functional recovery and rehabilitation, have been temporarily closed. Furthermore, the units devoted to musculoskeletal cancer surgery and to oncological and degenerative spine surgery were temporarily consolidated into a single unit. Outpatient services were also suspended.

One another note, during the pandemic period 126 healthcare professionals (out of a total of 1457), have been absent due to illness or diagnosis of Covid-19. In order to replace these absences, the healthcare professionals employed in those units whose activity was suspended were transferred to cover their ill colleagues of functioning units. The hospital management created also a department dedicated to the hospitalization of orthopaedic patients with confirmed/probable/suspected COVID-19 diagnosis. In this regard, one operating room (previously devoted to the management of infections of the musculoskeletal system) was converted to host surgeries of COVID positive patients. This reorganization led to an important flow of the nursing and physiotherapy staff within our hospital to guarantee pre-pandemic standards of care, but it was not always possible to consider the professionals’ specific clinical skills.

In addition, in three surgical units which were open during the pandemic period, temporary closures have been determined to contain the spread of COVID-19 hotspots among healthcare personnel and in-patients.

### Measures

The primary outcome was the incidence of any stage hospital-acquired PUs. PUs are one of the most frequent complications in hip fracture patients, ranging from 8.8% to 55% in the International literature [20], and an independent predictive factor of one-year mortality after surgery [21]. Moreover, several authors have identified hospital-acquired PUs as a major nurse-sensitive outcome [22]. Both PU definition and staging have been determined following the EPUAP classification [17]. The secondary outcomes included time (in days) required to achieve, assisted or independently, standing and walk for the first time. Moreover, data on overall hospital admission volume and in-hospital mortality for all types of surgery in the two groups (PP group and P group) were also collected. The following possible predictive factors for primary outcome, as identified by research team’s clinical experience and available literature [23–25], were also assessed:

-patient demographics and baseline characteristics (age, gender, pre-existing PUs at hospital admission, Braden score [16], hip fracture type;

-peri-operative care factors: time to surgery (days), type of surgical procedure (osteosynthesis vs. arthro- or endoprothesis surgery), use of anti-decubitus air mattress, insertion of a urinary catheter during hospitalization and length of time it remained in situ (days), changes of position while in bed of those expected (at least 4 daily), which were performed every 4 h, overall and pre and post-operative length of hospital stay (days), presence of a urinary catheter at hospital discharge.

All the above cited data were routinely recorded in the patient electronic clinical record by the Physician, the Registered Nurse, or the Physiotherapist on duty, as appropriate. For the present study, data (for both PP and P periods) were retrospectively extracted from the Institute’s computer-based patient-record system by a research Registered Nurse and a Physiotherapist.

### Statistical analysis

To compare the two groups (P group vs PP group), the χ2 test or Fisher's exact test (where appropriate) were employed for categorical variables; the Mann–Whitney test was applied for continuous variables. The relative risk of developing PUs was estimated using a log-binomial regression model. Considering the observational design of the study, two different analyses were performed to reduce confounding: one based on the log-binomial regression model, adjusted for Propensity Score (PS), and one based on the change-in-estimate.

The PS showed the probability of having been hospitalized between March and June 2020 and it was calculated using a logistic regression model including the following variables: gender, age at hospitalization, Braden score, number of days with catheter, fracture of the femoral neck, length of stay. The log-binomial regression model was then estimated by including the variable that identifies the pandemic group together with the PS.

As a sensitivity analysis, the log-binomial regression model was also estimated by a stepwise selection of the variables listed above, by including those that resulted in the largest change of the point estimate of the relative risk comparing the two cohorts (change-in-estimate approach). In detail, starting from the model estimate that included only the cohort variable, the variable that produced the largest change of the relative risk of the cohort comparison was added at each step in the model. When the inclusion of each additional variable produced a change of the relative risk of less than 5%, the selection procedure was stopped. All analyses were performed with Stata 11.2 (StataCorp, College Station, TX).

## Results

**Fig. 1:**
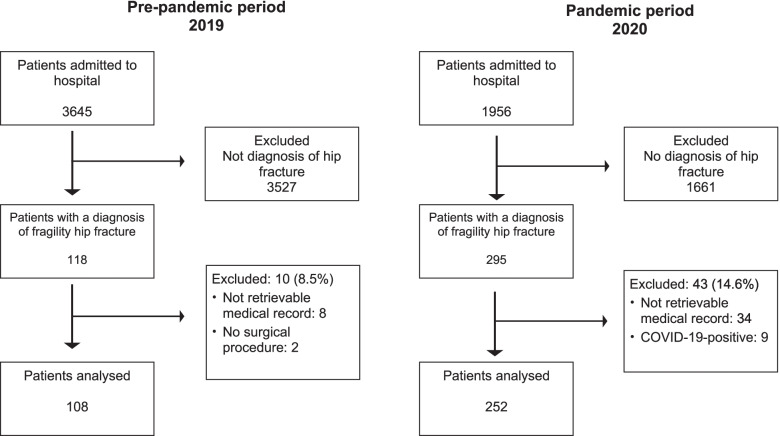
Flow Diagram

There were 360 patients who underwent hip fracture surgery in this study, which comprised 108 patients in PP group and 252 patients in P group. The patient flowchart is shown in Fig. 1. The increase in number of patients admitted with a hip fracture diagnosis was of 133% during the pandemic period compared with pre-pandemic period in 2019. On the contrary, overall inpatient hospital admissions decreased by 44% in pandemic period in 2020 (1956 patient admitted) compared to pre-pandemic period in 2019 (3645 patients admitted). The percentage of patients who underwent hip fracture surgery in PP group was almost 3%, without no significant month-to-month variation. On the contrary, during pandemic the rate of hip fracture patients surgically treated has changed over time with a percentage increase by 8.9% in March, 19.7% in April, 15.4% in May, 9.8% in June compared to 2019. Overall, in-hospital mortality rates (calculated on all admitted patients) increased from to 0.1% (*N* = 4) in the PP period to 0.9% (*N* = 17) in the P period (*p* < 0.001).

### Profile of hip fracture patients admitted during pandemic

Table 1 and 2 compares the patient demographics, peri-operative care factors and outcomes of the two groups. Comparing the sample’s characteristics in relation to the hospital admission period, the PP-group and P-group were homogeneous in the terms of the patient characteristics, with the exception of median age (82 years in PP-group vs 86 years in P-group; *p* = 0.006) and the type of fracture (femoral neck fracture 68.5% in PP-group vs 47.6% in P-group; *p* = 0.001).

**Table 1:** Patient demographics, baseline characteristics and peri-operative care in the pre-pandemic (PP) and pandemic (P) groups. Values are numbers (percentages) unless stated otherwise

	**PP (*n* = 108)**	**P (*n* = 252)**	**Overall (*n* = 360)**	**p-value**
**Patient demographics and baseline characteristics**				
Female gender, n (%)	81 (75.0)	199 (79.0)	280 (78.0)	0.41
Median (IQR) age, (years)	82 (12)	86 (10)	85 (11)	0.006
Presence of PUs at admission, n (%)	10 (9.3)	17 (6.8)	27 (7.6)	0.70
Patients with a femoral neck fracture, n (%)	74 (68.5)	120 (47.6)	194 (54.0)	0.001
Median (IQR) Braden Index score^a^	15 (2)	15 (2)	15 (2)	0.22
**Peri-operative care**				
Median (IQR) length of stay (days)	8.5 (4)	9.0 (6)	9.0 (5)	0.015
Median (IQR) pre-operative length of stays (days)	2 (1)	1 (1)	1 (1)	0.03
Median (IQR) post-operative length of stays (days)	7 (4)	7 (6)	7 (4)	0.004
Patients with an anti-decubitus air mattress, n (%)	97 (89.8)	209 (82.9)	306 (85.0)	0.094
Patients with a urinary catheter inserted in hospital, n (%)	76 (70.4)	173 (68.7)	249 (69.2)	0.75
Patients with a urinary catheter inserted in hospital or at home, n (%)	91 (84.3)	230 (91.3)	321 (89.2)	0.05
Patients with a urinary catheter at discharge, n (%)	41 (38.0)	94 (37.3)	135 (37.5)	0.91
Median (IQR) days with a urinary catheter	7 (4)	8 (5)	7 (5)	0.017
Median percentage (IQR) of changes of position while in bed of those excpected.	88 (11)	100 (8)	92 (17)	< 0.001
Patients with Osteosynthesis surgery, n (%)	46 (43.0)	133 (52.8)	179 (49.9)	0.074

*Pus* Pressure Ulcers, *IQR* interquartile range

^a^Braden Index score = from 6, severe risk, to 23, no risk of pressure ulcers

**Table 2:** Outcomes in the pre-pandemic (PP) and pandemic (P) groups. Values are numbers (percentages) unless stated otherwise

	**PP (*n* = 108)**	**P (*n* = 252)**	**Overall (*n* = 360)**	**p-value**
**Primary outcome**				
PUs incidence, n (%)	11 (10.2)	55 (21.8)	66 (18.3)	0.009
PUs anatomic location				0.004
Sacrum, n (%)	10 (90.9)	21 (38.2)	31 (47.0)	
Heel, n (%)	0 (0)	17 (30.9)	17 (25.8)	
Other locations, n (%)	1 (9.1)	17 (30.9)	18 (27.2)	
PUs stage				0.837
Stage I, n (%)	3 (30.0)	18 (33.3)	21 (32.8)	
Stage II, n (%)	7 (70.0)	36 (67.7)	43 (67.2)	
Median (IQR) time to PU development (days)	6 (3)	4.5 (6)	4.5 (4.7)	0.587
**Secondary outcomes**				
First standing, n (%)				0.37
≤ 2 days	59 (54.6)	144 (57.1)	203 (56.4)	
> 2 days	44 (40.7)	88 (34.9)	132 (36.7)	
Never	5 (4.7)	20 (8.0)	25 (6.9)	
Median (IQR) time to first standing (days)	2.0 (1.5)	2.0 (2.0)	2.0 (2.0)	0.34
First Ambulation, n (%)				0.65
≤ 3 days	57 (52.8)	120 (47.6)	177 (49.2)	
> 3 days	28 (25.9)	75 (29.8)	103 (28.6)	
Never	23 (21.3)	57 (22.6)	80 (22.2)	
Median (IQR) time to first ambulation (days)	3 (2)	3 (2)	3 (2)	0.59

With regard to peri-operative care factors, a statistically significant difference was found for some variables (Table 1). In particular, those patients admitted during the pandemic experienced a longer—overall and post-operative—in-hospital stay (*p* = 0.015 and 0.004, respectively) than those who were admitted in the pre-pandemic period. Moreover, they were more exposed to changes of position while in bed (88% in PP-group vs 100% in P-group; *p* < 0.001), and to the presence of a urinary catheter inserted both before and after hospital admission (84.3% in PP-group vs 91.3% in P-group; *p* = 0.05); although the number of catheters inserted during hospitalization was not significantly different between the two groups (*p* = 0.75).

Considering the primary outcome, a statistically significant difference was found in PUs incidence (10.2 in PP-group vs 21.8% in P-group; *p* = 0.009). The incidence of sacral PUs was significantly lower in P-group (38%) vs PP-group (91%) (*p* = 0.004); on the contrary, the incidence of PUs localized to the heels or other body sites were significantly higher in P-group (30% and 30%, respectively) vs PP-group (0% and 9%, respectively) (*p* = 0.004).

No statistically significant differences were found between groups for all the secondary outcomes (Table 2).

### Relative risk of pressure ulcers and predictive factors

**Fig. 2:**
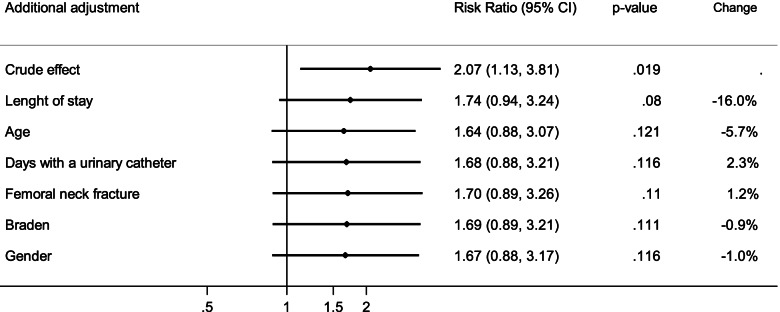
Change-in-estimate of Relative Risk for pressure ulcers incidence between pandemic period vs pre-pandemic period

From the analysis of the unadjusted risk ratio for the two periods taken under consideration, the results showed that the relative risk (RR) of pressure ulcers among patients admitted to hospital during the pandemic was 2.07 as compared to those patients admitted during the pre-pandemic period (*p* = 0.019) (Table 3). Analyses performed using the PS approach (adjusting for age, gender, Braden Index score, days with a urinary catheter, fracture type and length of stay) showed a reduction in the RR of PU comparing the two periods (RR = 1.63, *p* = 0.127). This evidence is also confirmed by adjusting the comparison using the change-in-estimate strategy. As shown in Fig. 2, the main unbalanced factors between the two periods that influenced the comparison were age and length of stay. Adjusting for these factors, the RR was nearly coincident with that estimated with the PS approach (RR = 1.64, *p* = 0.121). Further adjustment for the other factors produced negligible RR changes (< 5%).

**Table 3:** Effect of year of admission on risk of PU based on log-binomial model. Based on 351 patients with complete information for the calculation of propensity score ^a^

	**Risk Ratio**	**95% CI**	***p***-value
**Crude effect**			
2020 vs 2019	2.07	1.13–3.81	0.019
**Change-in-estimate adjustment**			
2020 vs 2019	1.64	0.88–3.07	0.121
Length of stay, per 1-day increase	1.07	1.03–1.10	< 0.001
Age, per 1-year increase	1.02	0.99–1.05	0.154
**Adjusted for Propensity score**^a^			
2020 vs 2019	1.63	0.87–3.05	0.127

^a^ Age, gender, Braden Index score, days with a urinary catheters, fracture site and length of stay

## Discussion

The COVID -19 pandemic forced the settings of an orthopaedic surgery hospital into a deep transformation of its organizational model, with a consistent modification of both its admission volumes and the most frequent type of surgery performed. The data of this research study highlight a 44% reduction in the number of admissions, with a simultaneous increase in the number of surgical operations due to fragility hip fracture. These results are confirmed by the findings of Wong et al. [26] in Hong Kong and by Hampton et al. [12] as regarding the English context, they both present similar trends.

From a perspective of “Nurse-Sensitive Indicators”, the results of our study indicate that the incidence of PUs in patients with fragility hip fractures increased notably: between the pre-pandemic period and the pandemic the incidence of this outcome went from 10 to 22%. As regarding the characteristics of the ulcers, no significant changes were noticed in terms of day of onset and lesions stage, on the contrary the PUs place of occurrence varied notably compared to the pre pandemic period. In 2019, 91% of all pressure ulcers were located in the sacrum area, however in 2020 the PUs incidence at the sacrum decreased to 38%, while the frequency of PUs located in other pressure points, such as heels, increased to 31%. Prior to the pandemic, Chiari et al. [25] explored the PUs incidence in a cohort with characteristics comparable to our current study population and reported a 64% incidence to the sacrum, 23% to the heels and 13% to other locations. Due to the small number of events recorded over the pandemic period, it hasn’t been possible to ascertain if the difference registered over this period may be related to the reorganization of the daily activities of clinical practice in many units or if it may be due to other factors. However, given the results of this study, we consider important to remind to all the health professionals that PUs prevention and monitoring cannot be focused only on the most commonly affected areas such as sacrum and heels, but they must include close monitoring of all body pressure points.

The unadjusted risk ratio for the development of pressure ulcers in relation to both the periods under exam, the pre-pandemic period in 2019 and the pandemic in 2020, has been found to be 2.07. The calculation of the relative risk ratio, adjusted for the significant risk factors emerged from the study, specifically age and length of stay, did not confirm the initial unadjusted score and its statistical significance. This demonstrates how these two risk factors have been the main responsible for the incidence variations found in the two periods.

In this regard, it is also interesting to notice that the difference of median age among the two cohorts is 4 years. The results of the present study indeed highlight age as a risk factor for the development of PUs and this is confirmed by multiple other studies [24, 25] in this patient population. In the same way, length of stay was identified as a risk factor for the development of Pus, confirming results of previous studies [27, 28]. On this matter it should also be considered that the pandemic has heavily affected the hospital length of stay of any patient category, not just COVID-19 patients, leading to difficulties in the organization of post-discharge rehabilitation pathways. Many outpatient rehab facilities and long-term care facilities either had to convert units or had to become hubs dedicated the treatment and care of COVID patients, therefore disrupting their usual capacity to receive orthopaedic patients discharged from the hospital. Furthermore, community medicine services were also heavily impacted by the high number of patients in need to be followed at home and by the new laws imposed by the Italian government in order to contain the spread of the virus.

Nevertheless, the analysis of the median time to PUs onset for the 2020 cohort showed similar timing to the pre-pandemic period, suggesting that patients tend to develop pressure ulcers during the first period/phase of their admission.

On the other hand, the investigation of other indicators of nursing sensitive outcomes, which have been previously found in the literature to be associated with pressure ulcers prevention, such as prolonged urinary catheterization, use of the anti-decubitus mattress, bed mobilization and early walking, did not underwent variations over the two periods examined. These findings allow us to assume that the quality of care received by the patients did not decrease during the pandemic period and that the healthcare workers continued to correctly apply the standard procedures for PUs prevention. These positive outcomes are confirmed also looking at the physiotherapy treatment administered to the patients over the pandemic period. The recovery of the ability to stand and to walk was achieved at an early stage, with an identical timing compared to 2019.

These results witness that the healthcare professionals were able to maintain and offer to the patients the same levels of care guaranteed prior to the pandemic, although under extremely stressful conditions. The qualitative evaluation of the care administered and the emotional impact of the pandemic on the patients are very interesting topic which would deserve further investigation.

### Limits

This is a retrospective study; however, the collected data have been accurately checked and they are exhaustive. Therefore, they allow an adequate statistical analysis of both the primary outcome and of the other rehabilitation and nursing sensitive outcomes. Currently in the literature there are not many studies containing data on nursing sensitive outcomes and consequently it has been difficult to compare our results with other institutions. On the other hand, one of the strengths of this study is the analysis of the data on the quality of the clinical care administered to the patients during the pandemic, with the objective to understand how institutions and health professionals reacted to the reorganization of the health services imposed by the global pandemic and how they performed, not just in the treatment of COVID-19 patients but also in the care of all hospitalized patients.

## Conclusion

Over the pandemic period, the clinical activities, as well as the nursing and physiotherapy care administered to fragility hip fracture patients maintained high standards of care, similar to the pre-pandemic standard of care. The development of pressure ulcers over the pandemic period increased, probably due to the older age of the patients admitted to the hospital.

## Data Availability

The datasets used and/or analysed during the current study are available from the corresponding author on reasonable request.

## Abbreviations

ICU: Intensive Care Unit
PP: Pre-pandemic
P: Pandemic
PU: Pressure ulcer
PS: Propensity Score
RR: Relative Risk
